# Phosphodiesterase 10A Is a Mediator of Osteogenic Differentiation and Mechanotransduction in Bone Marrow-Derived Mesenchymal Stromal Cells

**DOI:** 10.1155/2020/7865484

**Published:** 2020-06-06

**Authors:** Sigrid Müller-Deubert, Carolin Ege, Melanie Krug, Jutta Meißner-Weigl, Maximilian Rudert, Oliver Bischof, Franz Jakob, Regina Ebert

**Affiliations:** ^1^Bernhard Heine Center for Locomotion Research, Orthopedic Department, University of Würzburg, Friedrich-Bergius-Ring 15, 97076 Würzburg, Germany; ^2^Institute Pasteur, Department of Cell Biology and Infection, 25-28 Rue du Docteur Roux, 75015 Paris, France; ^3^INSERM U.993, 75015 Paris, France

## Abstract

Bone marrow-derived mesenchymal stromal cells (hMSCs) are capable of differentiating into the osteogenic lineage, and for osteogenic differentiation, mechanical loading is a relevant stimulus. Mechanotransduction leads to the formation of second messengers such as cAMP, cGMP, or Ca^2+^ influx resulting in the activation of transcription factors mediating gene regulation. The second messengers cAMP and cGMP are degraded by phosphodiesterase isoenzymes (PDE), but the role of these enzymes during osteogenic differentiation or mechanotransduction remains unclear. Here, we focused on the isoenzyme phosphodiesterase 10A (PDE10A) and its role during osteogenic commitment and mechanotransduction. We observed a time-dependent decrease of PDE10A expression in hMSC undergoing differentiation towards the osteogenic lineage. PDE10A inhibition by papaverine diminished osteogenic differentiation. While applying mechanical strain via cyclic stretching of hMSCs led to an upregulation of PDE10A gene expression, inhibition of PDE10A using the drug papaverine repressed expression of mechanoresponsive genes. We conclude that PDE10A is a modulator of osteogenic differentiation as well as mechanotransduction in hMSCs. Our data further suggests that the relative increase of cAMP, rather than the absolute cAMP level, is a key driver of the observed effects.

## 1. Introduction

Bone is a complex tissue that is formed by mesoderm-derived stem cells during development. In adult organisms, it is maintained, repaired, and remodeled by skeletal and endothelial precursors residing in bone and the bone marrow [1–3]. Core osteogenic signaling cascades orchestrate these processes, e.g., the wnt/frizzled pathway, the TGF*β*-related family of receptors with bone morphogenetic proteins (BMPs) as ligands, and type 1 parathyroid hormone receptor (PTH1R) signaling as induced by parathyroid hormone (PTH) and PTH-related peptide (PTHLH). The latter represents an important pathway that is linked to the production of the second messengers cyclic AMP (cAMP) and cyclic GMP (cGMP). The PTH1R ligand/receptor complex activates, e.g., adenylate cyclase and protein kinase A. Subsequently, the cAMP-response element-binding protein (CREB) is phosphorylated and binds to respective CRE elements in the promoters of target genes like c-fos and c-jun, RUNX2 (runx-related transcription factor 2), and BMPR2 (bone morphogenetic protein receptor II). All these factors support bone formation and fracture healing [4, 5].

Mechanical strain is also an important cue to stimulate bone formation for adaptation to environmental needs [6]. Mechanotransduction in skeletal precursor cells has been described to be cAMP-dependent and regulated by adenylyl cyclase 6 at the microdomain of the primary cilium [7]. A second cascade involving cGMP/protein kinase G signaling as a mechanoresponsive pathway in osteoblasts has also been described [8]. Mechanotransduction is, besides the abovementioned signaling pathways, a potent stimulus for osteogenic differentiation and bone formation. Sclerostin is an osteocyte-secreted potent inhibitor of osteogenic WNT signaling, and its gene *SOST* is addressed by physical forces [9]. For example, exercise, inhibits sclerostin production and creates a permissive environment for bone formation and repair [10, 11]. A second regulatory stimulus comes from ligand activated PTH1R osteogenic signaling. Sclerostin production is also downregulated through intermittent PTH signaling. The efficacy of PTH-induced bone formation again strongly depends on mechanical loading, thereby introducing a second level of control by mechanotransduction [12, 13].

The regulation of cAMP and cGMP as second messengers is controlled by the balance between the activity and the subcellular distribution of respective cyclases and their antagonists, the inactivating enzyme families of phosphodiesterases. The mechanisms of generation and downstream signaling have been extensively explored in general and also in bone-related signaling cascades. However, less is known about the role of intracellular cAMP/cGMP antagonists that fine-tune or blunt these signals, like members of the phosphodiesterase (PDE) protein family [14]. In the case of PTH signaling, for example, a signaling cascade involving *β*-arrestin recruitment induces PDE4 as an important target. In a limited number of studies, the mildly positive influence of PDE inhibitors, like the PDE4 inhibitor rolipram and the PDE5 inhibitors avanafil and sildenafil, on bone maintenance, formation, and fracture healing has already been described [15–17]. The general function of phosphodiesterases is well known. They hydrolyze cyclic AMP and cyclic GMP second messenger molecules, and eleven families with variable specificity for cAMP and cGMP were described [14]. PDEs vary in their subcellular distribution, and together with kinases such as protein kinase A and with respective anchoring proteins like A kinase anchoring proteins (AKAPs), they modulate the efficacy of kinase-dependent activation of transcription factors. PDEs are involved in many relevant signaling pathways in cardiovascular and neuronal systems as well as in cancer. One of the best known examples is the PDE5 family, the inhibition of which modulates vasoconstriction [14, 18, 19].

Phosphodiesterase 10A (PDE10A) is mainly expressed in the brain, especially in neuronal cells of the striatum, with many different splice variants, and is associated with schizophrenia and neurodegenerative diseases [14, 20, 21]. The isoenzyme PDE10A has an almost 80-fold higher affinity towards cAMP (Km 56 nM) compared to the affinity of cGMP (Km 4.4 *μ*M) [22]. In neurons, PDE10A is involved in the regulation of dopaminergic and glutamatergic signaling and treatment with the specific inhibitor papaverine leads to a marked increase of cAMP and cGMP levels [23]. PDE10A knockout animals show lower body weight than their wild-type siblings while female animals were more affected than males [24]. Consistent with the differences in body weight, PDE10A is important for the regulation of energy balance of brown and white fat cells as well as insulin resistance [25, 26]. Knockout mice also presented with decreased locomotor activity and exploratory behavior in new surroundings.

No information is available about gene regulation of PDE10A and its role in mechanotransduction and osteogenic differentiation. Here, we report on PDE10A expression profiling in skeletal precursor cells isolated from femoral heads from a patient cohort undergoing hip replacement therapy. PDE10A is mechanosensitive in skeletal precursors, and its upregulation may impair the osteogenic signals as discussed above. Our data strongly suggest that this mechanoresponsive gene is relevant for mesenchymal stromal cell biology and osteogenic differentiation and may play a role in bone regeneration.

## 2. Materials and Methods

### 2.1. Cell Culture

Media for cell culture were obtained from Thermo Fisher Scientific (Darmstadt, Germany), FCS was obtained from Bio&Sell GmbH (Ulm, Germany) [27]. Primary human mesenchymal stromal cells (hMSCs) were isolated from the bone marrow from different donors and cultivated up to four weeks by a standardized protocol [28]. Bone marrows were recovered after informed consent from the explanted femoral heads of patients undergoing elective hip arthroplasty. The procedure was approved by the local Ethics Committee of the University of Würzburg (186/18). Briefly, bone marrow preparations were washed with Dulbecco's modified Eagle's medium, (DMEM/F12) supplemented with 10% FCS, 100 U/ml penicillin, 0.1 mg/ml streptomycin, and 50 *μ*g/ml ascorbate (Sigma-Aldrich GmbH, Munich, Germany), and centrifuged at 1200 rpm for 5 min. The pellet was reconstituted in medium and washed four times, and the supernatants of the washing steps containing the released cells were collected. Cells were centrifuged and cultivated at a density of 1 × 10^9^ cells per 175 cm^2^ culture flask. Adherent cells were washed after 2 days and cultivated until confluence. MSCs from mice were isolated from calvariae by using an established protocol. Briefly, calvariae were prepared and collected in PBS in a 15 ml tube. PBS was removed, and digestion solution (*α*-MEM containing 0.1% collagenase type Ia (Sigma-Aldrich GmbH) and 0.2% dispase grade II (Roche)) was added and incubated on a shaker for 20 min at 37°C. The cell-containing supernatant was transferred to a new 15 ml tube, and again, 2 ml of digestion solution was added, followed by a 20 min incubation period at 37°C on a shaker. Cells were again transferred to the collection tube, pelleted at 1200 rpm for 3 min, resuspended in 2 ml culture medium (*α*-MEM supplemented with 10% FCS and 100 U/ml PenStrep), and seeded on a 35 mm dish. MC3T3 preosteoblasts were cultivated in *α*-MEM plus nucleosides, supplemented with 10% FCS and 100 U/ml PenStrep. SH-SY5Y neuroblastoma cells were cultivated in Dulbecco's modified Eagle's medium (DMEM/F12), supplemented with 10% FCS and 50 *μ*g/ml gentamycin (Sigma-Aldrich GmbH). All cells were grown at 37°C in a humidified atmosphere consisting of 5% CO_2_ and 95% air.

### 2.2. Preparation of Murine Brain Lysates

Whole brains from mice were prepared, shock frozen in liquid nitrogen, and pulverized with a micro dismembrator S (Sartorius). One-third was transferred to a new tube, and total RNA was isolated by using the NucleoSpin RNA II kit (Macherey-Nagel, Düren, Germany) according to the manufacturer's instructions.

### 2.3. Cell Viability and Apoptosis Assays

To determine effects of the specific PDE10A inhibitor papaverine hydrochloride [29–31] (Sigma-Aldrich GmbH, Schnelldorf, Germany) on viability and apoptosis, human MSCs were seeded on a 96-well plate with a density of 1000 cells/well and were treated with 1, 10, and 100 *μ*M papaverine for 24, 48, and 72 h. Viability and apoptosis rates were assessed using the CellTiter-Glo Luminescent Cell Viability Assay and the Caspase-Glo 3/7 Assay, respectively, (both from Promega GmbH, Mannheim, Germany), according to the manufacturer's instructions. Luminescence was measured with an Orion II Luminometer (Berthold Detection Systems, Pforzheim, Germany). Data are expressed as mean from triplicates of three independent donors.

### 2.4. Cyclic Stretching of hMSC

For cyclic stretching, 5 × 10^5^ cells per well were seeded on 4-well PU plates as described previously [32, 33] and cultivated for one week overall. 10 *μ*M papaverine was added for 24 hours, and 1 mM db-cAMP (N6,2′-O-dibutyryl-cAMP, Sigma-Aldrich GmbH, Schnelldorf, Germany) was added 1 h before stretching. The respective db-cAMP concentration was obtained from [34]. Cells were mechanically stimulated by applying 1 Hz with an extension of 1% for 30 min using a bioreactor and polyurethane dishes developed by our group [35]. Cells were harvested after 15 min and 4 h, respectively, and total RNA was isolated by using the NucleoSpin RNA II kit (Macherey-Nagel, Düren, Germany) according to the manufacturer's instructions.

### 2.5. Osteogenic Differentiation of hMSC

Human MSCs were differentiated into the osteoblastic lineage by seeding 1 × 10^4^ cells per cm^2^ in 6-well plates for RNA isolation and for histochemical staining. After reaching confluence, the medium was replaced by osteogenic medium consisting of DMEM High Glucose, 10% FCS, 1 U/ml penicillin, 100 *μ*g/ml streptomycin (all Life Technologies GmbH), 50 *μ*g/ml L-ascorbic acid 2-phosphate, 1 *μ*M dexamethasone, and 10 mM *β*-glycerophosphate (all from Sigma-Aldrich GmbH). Control cells were kept in expansion medium. Influence of papaverine hydrochloride and db-cAMP on mineralization and gene expression was tested by adding 10 *μ*M papaverine and 1 mM db-cAMP throughout the osteogenic differentiation process. The medium was changed twice a week.

### 2.6. Histochemical Staining

For the detection of calcium hydrogen phosphate and hydroxylapatite in the extracellular matrix, hMSCs were fixed in methanol after 2 weeks, stained with alkaline alizarin red S (1% *w*/*v*) (Chroma-Schmidt GmbH, Stuttgart, Germany) for 2 min, and air dried. Microscopic images were taken at room temperature with an Axio Observer 7 microscope with a 10x/0.3 Plan Neofluar objective and an Axiocam 305 camera (all from Carl Zeiss Microimaging GmbH, Göttingen, Germany).

### 2.7. Isolation of RNA, RT-PCR, and Quantitative PCR

Total RNA was isolated by using the NucleoSpin RNA II kit (Macherey-Nagel, Düren, Germany) according to the manufacturer's instructions. One microgram of total RNA was reverse-transcribed with MMLV reverse transcriptase (Promega GmbH) in a volume of 25 *μ*l. For quantitative PCR, the cDNA was diluted 1 : 10 and qPCR was performed in 20 *μ*l by using 2 *μ*l of cDNA and 10 *μ*l of the GoTaq qPCR Master Mix (Promega GmbH, Mannheim, Germany) and 0.25 pmol of sequence-specific primers obtained from biomers.net GmbH (Ulm, Germany) or Qiagen GmbH (Hilden, Germany) (see Table 1 for primer sequences and PCR conditions). qPCR conditions were as follows: 95°C for 3 min; 40 cycles: 95°C for 10 s; annealing temperature (see Table 1) for 10 s; 72°C for 10 s; followed by melting curve analysis for specificity of qPCR products. Results were calculated with the efficiency-corrected Ct model [36] with RPS27A (Ribosomal Protein S27a) as the housekeeping gene. Data were obtained from three to four independent experiments, and qPCRs were performed three times with the qPCR thermal cycler qTOWER^3^ (Analytik Jena AG, Jena, Germany). Differences were calculated with the *ΔΔ*CT method; significances were obtained with the Student *t*-test.

### 2.8. Western Blot

After osteogenic differentiation of hMSC for different time points with and without 10 *μ*M papaverine, cells were washed twice with PBS and harvested in 200 *μ*l lysis buffer (50 mM HEPES, 150 mM NaCl, 5 mM EDTA, 0.1% NP-40, 20 mM *β*-glycerophosphate, and 0.5 mM Na-orthovanadate, containing cOmplete™ proteinase inhibitor (Roche Diagnostics Deutschland GmbH, Mannheim, Germany)). Cells were lysed by sonification followed by centrifugation at 10000 rpm, 4°C for 10 min.

Proteins were quantified by RotiQuant assay (Carl Roth GmbH, Karlsruhe, Germany). 15 *μ*g protein was mixed with 3.75 *μ*l loading buffer (RotiLoad, Carl Roth GmbH) and denatured by boiling for 5 min. Samples were separated on 12% SDS polyacrylamide gels, 375 mM Tris (pH 8.8), 0.1% SDS in 192 mM glycine, and 25 mM Tris, 0.1% SDS (pH 8.8). After SDS PAGE, the gel was incubated with CAPS buffer (50 mM CAPS, 10% methanol, and 1 mM mercaptopropionic acid, pH 10.0) for 10 min. Proteins were semidry blotted for 1 h at 1 mA/cm^2^ at room temperature to a stabilized nitrocellulose membrane (Optitran BA-S, Schleicher and Schuell, Dassel, Germany) using a Mini Protean unit (BioRad, München, Germany). Membranes were blocked with 5% nonfat milk powder or 5% BSA in TTBS buffer (0.1 M Tris, 150 mM NaCl, and 0.1% Tween-20), respectively, and incubated with PDE10A (G-7, mouse mAB, sc-515023, 1 : 500, Santa Cruz Biotechnology, Heidelberg, Germany) and *β*-actin (13E5 Rabbit mAB, #4970, 1 : 1,000, Cell Signaling, Leiden, the Netherlands) primary antibodies, respectively, diluted in blocking solution overnight at 4°C. Membranes were washed 3× for 15 min with TTBS followed by an incubation for 2 h at room temperature with mIgG*κ* BP-HRP (sc-516102) and the secondary anti-rabbit IgG-horseradish peroxidase antibody (SH A0545 Sigma-Aldrich GmbH), respectively, diluted 1 : 2000 in TTBS solution. After another washing for 3× for 15 min with TTBS, specific staining was detected using the chemiluminescence (ECL) system (VWR International GmbH, Darmstadt, Germany). Experiments were performed with three independent donors. One representative experiment is shown.

### 2.9. Statistical Analyses

Statistical analyses were performed using two-tailed unpaired or paired *t*-test, and *p* values less than 0.05 were considered significant. All values were obtained from at least three technical replicates and expressed as mean ± SEM. Asterisks indicate significant differences against control samples used for normalization. Further details of number of independent experiments, hMSC donors used, and selection of the normalization method are given in the figure legends.

## 3. Results

### 3.1. PDE10A Is Expressed in Human and Murine Primary MSCs and Cell Lines

**Figure 1:**
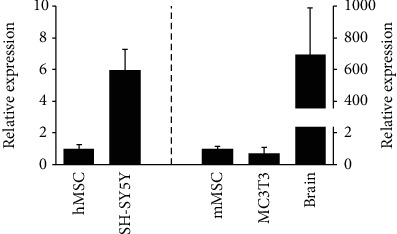
PDE10A expression in human primary MSC (hMSC, *n* = 7), in human neuroblastoma SH-SY5Y cells (*n* = 9), in murine primary MSC (mMSC, *n* = 3), in murine MC3T3 cells (*n* = 3), and in murine brain lysates (*n* = 3) is shown. Murine MSC and brain lysates were prepared from the identical mice. RPS27A (human samples) and B2m (murine samples) were used as housekeeping genes. QPCR data were obtained from technical triplicates, and results are shown as mean ± SEM; fold change was calculated with the *ΔΔ*CT method.

To determine *PDE10A* transcript levels in human and murine primary MSC and cell lines, we measured its gene expression by qPCR. *PDE10A* mRNA was detectable, both in human primary MSC and primary MSCs isolated from murine calvariae (Figure 1) as well as murine preosteoblastic cell line MC3T3. We used human neuroblastoma cell line SH-SY5Y and murine brain lysates as positive controls for their renowned high levels of PDE10A expression.

### 3.2. Osteogenic Differentiation Inhibits the Expression of PDE10A

**Figure 2:**
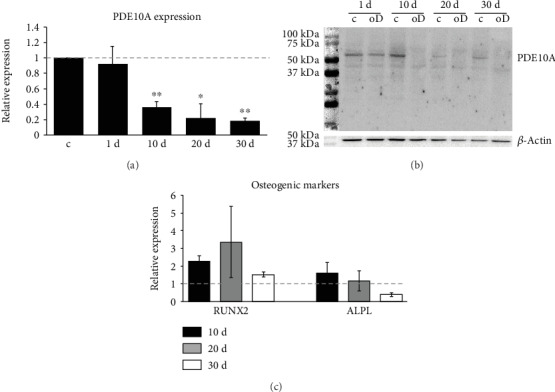
PDE10A expression in hMSC after osteogenic differentiation. QPCR (a) and Western blot (b) analysis of PDE10A as well as the expression of the osteogenic markers RUNX2 and ALPL (c) in primary human MSC (*n* = 4) differentiated towards the osteogenic lineage for different time points as indicated. RPS27A was used as the housekeeping gene; *β*-actin was used as loading control. QPCR data were obtained from technical triplicates, and results are shown as mean ± SEM. Fold change was calculated with the *ΔΔ*CT method. Significances were calculated with the Student *t*-test (^∗^*p* < 0.05; ^∗∗^*p* < 0.005). A representative Western blot is shown. ALPL: tissue nonspecific alkaline phosphatase; c: control; oD: osteogenic differentiation; PDE10a: phosphodiesterase 10a; RUNX2: runt-related transcription factor 2.

Human primary MSCs were cultivated to confluency and differentiated towards the osteogenic lineage. Total RNA and whole-cell lysates were prepared from cells harvested after 1, 10, 20, and 30 days, and the expression of *PDE10A* was analyzed by qPCR and Western blot. Both PDE10A gene expression and protein level were diminished after 10, 20, and 30 days of osteogenic differentiation when compared to the undifferentiated control (*p* < 0.05 and *p* < 0.005, Figure 2). To confirm that MSC developed towards the osteoblastic phenotype, we measured transcription factor *RUNX2* (runt-related transcription factor 2) and early osteoblastic marker *ALPL* (tissue nonspecific alkaline phosphatase) expression at indicated time points after differentiation. A trend could be appreciated in the expression of *RUNX2*, which was increased after 10 and 20 days, and *ALPL*, which was slightly enhanced after 10 days, due to high donor variabilities.

### 3.3. PDE10A Inhibition Does Not Affect Cell Viability and Apoptosis Rates of hMSCs

**Figure 3:**
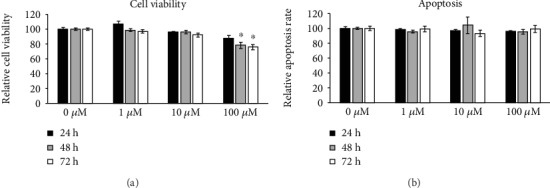
Effect of papaverine on viability and apoptosis of hMSCs derived from four donors. Cells were treated with 0, 1, 10, and 100 *μM* papaverine, and viability (a) and apoptosis assays (b) were performed 24, 48, and 72 h later. Relative luminescence is given. Data are expressed as mean of two independent experiments ± SEM and normalized to untreated control. Each measurement was performed in technical triplicate. Student's *t*-test was used for statistical analysis (^∗^*p* < 0.005).

In a next step, we blocked PDE10A activity by using the specific inhibitor papaverine in hMSCs. To identify the optimal concentration for PDE10A inhibition without affecting cell viability, hMSC were treated with 1, 10, and 100 *μ*M papaverine. Cell viability and the apoptosis rate were determined after 24, 48, and 72 h, respectively. While papaverine did not induce apoptosis, it diminished cell viability at 100 *μ*M after 48 and 72 h (*p* < 0.005, Figures 3(a) and 3(b)). Therefore, in all other experiments, 10 *μ*M papaverine for PDE10A inhibition was used as with this dose, no effects on cell viability were observed.

### 3.4. Inhibition of PDE10A Impairs Osteogenic Differentiation of hMSC

**Figure 4:**
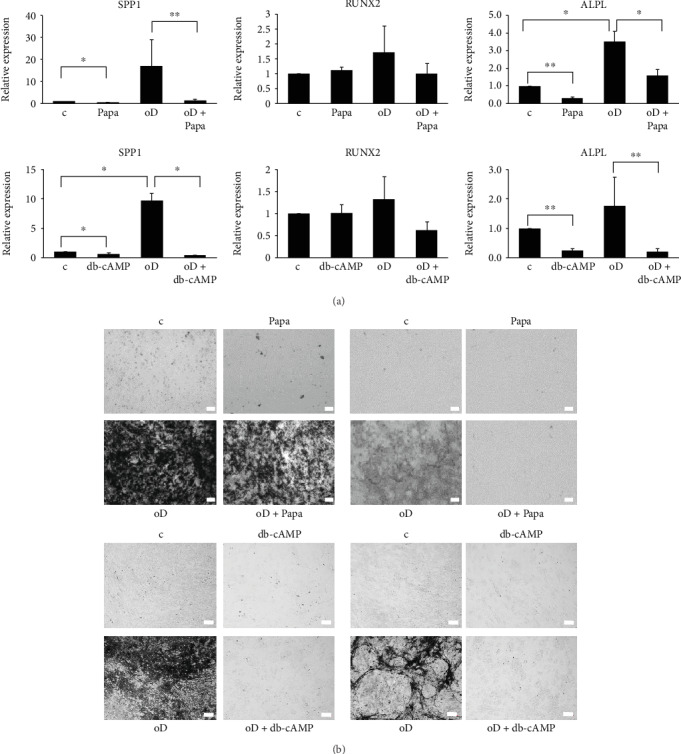
Analysis of osteogenic differentiation in primary human MSC (*n* = 5). (a) QPCR analyses of SPP1, RUNX2, and ALPL expression after 14 days of osteogenic differentiation and respective controls with and without 10 *μ*M papaverine or 1 mM db-cAMP. RPS27A was used as the housekeeping gene. QPCR data were obtained from technical triplicates and results are shown as mean ± SEM. Fold change was calculated with the *ΔΔ*CT method. Significances were calculated with the Student *t*-test (^∗^*p* < 0.05; ^∗∗^*p* < 0.005). (b) Alizarin red staining of primary MSC differentiated towards the osteogenic lineage for 14 days with and without application of 10 *μ*M papaverine or 1 mM db-cAMP. Four representative donors are shown. The bar represents 100 *μ*m. ALPL: tissue nonspecific alkaline phosphatase; c: control; db-cAMP: dibutyryl-cAMP; oD: osteogenic differentiation; Papa: papaverine; RUNX2: runt-related transcription factor 2; SPP1: secreted phosphoprotein 1.

To analyze if PDE10A is relevant for osteogenic differentiation, primary MSCs were differentiated towards the osteogenic lineage for 14 days and the specific PDE10A inhibitor papaverine or db-cAMP was added to the osteogenic cocktail, respectively. After 14 days of differentiation, the osteogenic marker genes *SPP1* (secreted phosphoprotein 1), *RUNX2*, and *ALPL* were analyzed by qPCR as well as mineral deposition by alizarin red staining. As expected, osteogenic marker genes were increased after osteogenic differentiation. While *RUNX2* was only slightly upregulated and with high donor variability, *ALPL* and *SPP1* were significantly enhanced. By contrast, the addition of papaverine or db-cAMP prevented the osteogenic upregulation of *RUNX2*, *SPP1* (*p* < 0.05), and *ALPL* (*p* < 0.05). Papaverine or db-cAMP also showed effects in undifferentiated cells and reduced the expression of *SPP1* and *ALPL* (*p* < 0.05 and *p* < 0.005, Figure 4(a)). The inhibition of osteogenic differentiation by papaverine or db-cAMP could also be verified by alizarin red staining. After 14 days of differentiation, mineral deposition was detectable in primary MSC, which was largely diminished in the presence of papaverine and db-cAMP, respectively (Figure 4(b)).

As dexamethasone (Dex) is a component of the osteogenic differentiation cocktail, we investigated if PDE10A was Dex responsive in hMSC and therefore treated hMSC with 1 *μ*M Dex but no effect on PDE10A mRNA expression could be detected (Suppl. Figure 1). In addition, papaverine did not influence the expression of PDE10A (Suppl. Figure 1).

### 3.5. PDE10A Is a Mechanoresponsive Gene in MSC

**Figure 5:**
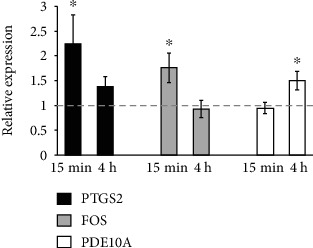
Gene expression after cyclic stretching of primary MSCs derived from 13 donors. Relative mRNA expression of PDE10A and the mechanoresponsive genes FOS and PTGS2 15 min and 4 h after mechanical loading. QPCR data were obtained from three independent qPCR experiments. Results are shown as mean ± SEM; fold change was calculated with the *ΔΔ*CT method and normalized to basal activity (nonstretched, dashed line). RPS27A served as the housekeeping gene. Significances were calculated with the Student *t*-test (^∗^*p* < 0.05). FOS: fos protooncogene; PDE10A: phosphodiesterase 10A; PTGS2: prostaglandin-endoperoxide synthase 2.

Mechanical strain is a key stimulus for osteogenic differentiation. To clarify if PDE10A is specifically responsive to mechanical strain in osteogenic precursors, human primary MSCs from 13 donors were seeded on polyurethane (PU) dishes and mechanical strain (1 Hz frequency, 1% extension) was applied. Whereas expression of PDE10A was increased 4 h after mechanical loading (*p* < 0.05; Figure 5, white bars), no such increase was observed early after 15 min. To confirm that primary MSCs are mechanosensitive and respond to cyclic stretching, we measured the expression of the mechanoresponsive genes, *PTGS2* (prostaglandin-endoperoxide synthase 2) and *FOS* (fos protooncogene) [38, 39] in the same donors 15 min and 4 h after mechanical loading (Figure 5, black and grey bars). In contrast to *PDE10A*, *PTGS2* and *FOS* were induced early after 15 min (*p* < 0.05) and the expression of PTGS2 and FOS declined to baseline 4 h after cyclic stretching, as we have reported before [33]. RPS27A was used as the housekeeping gene, which we used in former studies [33] and was not responding to cyclic stretching (data not shown). QPCR revealed time-dependent induction of PTGS2, FOS, and PDE10A while all donors responded to cyclic strain and were mechanoresponsive.

### 3.6. Inhibition of PDE10A Modulates Mechanoresponse in MSC

**Figure 6:**
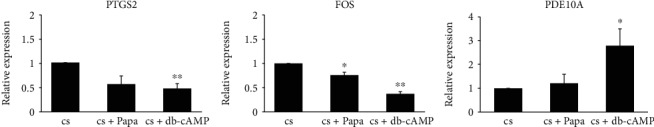
Effect of PDE10A inhibition and cAMP stimulation on mechanotransduction in primary MSC. Relative mRNA expression of the mechanoresponsive genes PTGS2 and FOS 15 min after cyclic stretching and PDE10A 4 h after cyclic stretching. Cells were pretreated with 10 *μ*M papaverine for 24 h or pretreated with 1 mM db-cAMP for 1 h. Results are shown as mean of five independent experiments by using five different MSC donors ± SEM and normalized to the values obtained from stretched samples. Fold change was calculated with the *ΔΔ*CT method, and RPS27A served as the housekeeping gene. Significances were calculated with the Student *t*-test (^∗^*p* < 0.05; ^∗∗^*p* < 0.005). cs: cyclic stretching; db-cAMP: dibutyryl-cAMP; FOS: fos protooncogene; Papa: papaverine; PDE10A: phosphodiesterase 10A; PTGS2: prostaglandin-endoperoxide synthase 2.

We have shown that PDE10A inhibition by papaverine causes the repression of the osteogenic marker genes *SPP1*, *RUNX2*, and *ALPL* and that PDE10A expression is induced after mechanical strain. Therefore, we asked if PDE10A inhibition has an impact on mechanotransduction in primary MSCs. To this end, we seeded cells of five different donors in PU dishes and pretreated them with 10 *μ*M of the PDE10A inhibitor papaverine for 24 h. Additionally, we used 1 mM db-cAMP for 1 h to increase intracellular cAMP concentrations. Subsequently, we applied mechanical strain (1 Hz frequency, 1% extension) and analyzed the expression of the mechanoresponsive genes *PTGS2* and *FOS* after 15 min, as well as *PDE10A* after 4 h, by qPCR. The mechanoresponse of PTGS2 and FOS was diminished by db-cAMP (*p* < 0.005; Figure 6). Papaverine pretreatment reduced the mechanoresponse of FOS (*p* < 0.05), and a trend could be appreciated in the downregulation of PTGS2. No effect of papaverine could be seen on the expression of *PDE10A* in this context. By applying db-cAMP, the mechanoresponse of PDE10A was increased by the factor 2.5 (*p* < 0.05).

## 4. Discussion

In this paper, we analyzed the expression and regulation of phosphodiesterase 10A (PDE10A) during osteogenic differentiation and mechanotransduction in primary human bone marrow-derived mesenchymal stromal cells (hMSCs). PDE10A belongs to the family of PDE isoenzymes, which degrade second messengers by hydrolyzing the 3′ cyclic phosphate bonds of cAMP and cGMP. The isoenzyme PDE10A exhibits an almost 80-fold higher affinity towards cAMP (Km 56 nM) compared to the affinity reported for cGMP (Km 4.4 *μ*M) [22], and therefore, we hypothesize that the here discussed results are largely dependent on modulating cAMP concentrations, while cGMP plays only a minor role.

In neurons, PDE10A is involved in the regulation of dopaminergic and glutamatergic signaling [23]. As PDE10A is well known to be expressed in neuronal tissues, we used murine brain lysates and the neuroblastoma cell line SH-SY5Y as positive controls in qPCR experiments. PDE10A mRNA expression could be detected in hMSC, murine primary MSC (mMSC), and the murine preosteoblastic cell line MC3T3, although to a much lesser extent compared to the expression in neuronal cells and tissues. Ahlstrom et al. investigated the expression of 15 different PDE family members and subtypes in SaOS-2 and MG-63 osteosarcoma as well as normal human osteoblastic (NHOst) cells, but PDE10A was only present in MG-63 cells [40, 41]. Therefore, our paper contributes to the existing reports and extends the knowledge about the expression and function of PDE10A in human bone marrow-derived stromal cells and osteogenic precursors.

Differentiation of hMSC towards the osteogenic lineage revealed time-dependent downregulation of PDE10A mRNA and protein. For dexamethasone (Dex), it was reported that it inhibits the expression of PDE family members, including PDE10A [41]. We hypothesize that the decrease of PDE10A expression during osteogenic commitment is an effect of the differentiation process itself as we did not observe dexamethasone effects in hMSC. Our data suggest that cAMP levels are low in the initial phase of osteogenic commitment, due to a higher expression of PDE10A, and are increased during osteogenic commitment due to a decrease of PDE10A expression. This is in line with the PDE10A expression pattern in osteosarcoma cells mentioned above, where MG-63, negative for ALP and less mature, are positive for PDE10A, and SaOS-2 cells, positive for ALP and more mature, are negative for PDE10A [41, 42]. In murine MSC and embryonic stromal cells, it was shown that during the early phase of osteogenic differentiation, the activation of adenylate cyclase via forskolin treatment led to an inhibition of mineralization [43], while generally lowering cAMP concentrations, by stimulating cells with 2′,5′-didesoxyadenosine, increased osteogenic differentiation [44]. In contrast, it was shown that pretreating MSC with forskolin before adding the osteogenic differentiation cocktail increased their osteogenic potential [43]. Enhanced osteogenic differentiation capacity was also reported for murine ST-2 stromal cells after selectively inhibiting PDE2, PDE3, and PDE4, which could not be verified in preosteoblastic MC3-T3 cells [45]. These reports indicate that fine-tuning of cAMP levels by PDE isoenzymes is relevant for the early phase of osteogenic commitment although their role during osteogenic differentiation is not fully understood.

As none of the here mentioned papers focused on the function of PDE10A, we intended to decipher its role during osteogenic differentiation by using a specific PDE10A inhibitor, namely, papaverine. Papaverine (1-(3,4-dimethoxybenzyl)-6,7-dimethoxyisoquinoline), the only clinically used PDE10A inhibitor so far, has an IC50 value of 36 nM for PDE10A and was described as highly specific for PDE10A [29, 46]. We excluded cytotoxic effects of papaverine at concentrations up to 10 *μ*M and used this dosage for all other experiments. PDE10A inhibition blunted the expression of osteogenic markers and reduced the mineralization rate of hMSC under osteogenic conditions, as did the stimulation with high nonphysiological cAMP levels obtained upon db-cAMP treatment. As mentioned above, in ES cells, high levels of cAMP during the early phase of osteogenic commitment are disadvantageous [44] and this is in line with our observations. Additionally, it was shown in rodent models that signaling via cAMP and protein kinase A inhibits osteogenic differentiation and bone formation [47] and that intermittent cAMP accumulation is correlated to an inhibition of osteogenesis [34]. However, the same group reported that high nonphysiological concentrations of cAMP—which in their opinion cannot be achieved by stimulating cells with natural GPCR ligands as, for example, PTH—are beneficial for osteogenic differentiation of human MSCs [48]. The same holds true for a continuous rise in cAMP levels or the continuous activation of PKA with db-cAMP [34]. The authors summarize that cAMP can exert a positive as well as a negative effect on osteogenic differentiation, and they conclude that not the cAMP concentration itself was the responsible trigger for cell fate decision but the respective time frame of application and duration [34]. We intended to determine cAMP levels after treating hMSC with papaverine by using two different commercially available cAMP assays. One was an immunobased assay, and the other one determined cAMP levels by measuring the activity of protein kinase A. We were unable to detect cAMP levels or cAMP variations in hMSC due to a lack of sensitivity of the used assays. Nevertheless, our data presented in this paper support the hypothesis that the regulation and fine-tuning of cAMP levels are crucial for osteogenic commitment and that PDE10A is a main player in this context.

Mesenchymal fate decision and especially osteogenic differentiation are also influenced by mechanotransduction following, e.g., cyclic stretching or fluid flow. Mechanical forces are translated into biochemical signals via integrins and calcium channels associated to the cell membrane and the primary cilium [6, 49]. Mechanotransduction results in the nuclear translocation and activation of transcription factors that bind to mechanoresponsive DNA elements, e.g., AP-1, SP-1 sites, or other shear stress DNA-response elements [35, 50, 51]. In MSC, it was shown that mechanotransduction is sensed by the primary cilium and regulated by adenylyl cyclase 6 and the second messenger cAMP [7]. Fluid shear stress is also mediated via cGMP in osteoblastic cells leading to the activation of cGMP-dependent protein kinase [52]. As PDE10A is involved in the degradation of cAMP and to a lesser extent of cGMP, we investigated if PDE10A is mechanoresponsive in hMSC. As we have reported before, the immediate-early genes *FOS* and *PTGS2* are upregulated within 15 min after the application of mechanical strain [33]. After 4 h, we found an upregulation of *PDE10A* expression in these cells, suggesting that the second messenger cAMP, which might be responsible for the upregulation of *FOS* and *PTGS2*, is degraded by PDE10A. We hypothesize that not only during osteogenic differentiation but also in mechanotransduction the time-frame or duration of cAMP signaling is important for the activation of signaling cascades. Papaverine blunted the mechanical load-driven upregulation of FOS and PTGS2. This suggests that cAMP increases through the inhibition of its catabolism and mechanotransduction-related cAMP increase, cooperate, and may even replace each other at least as far as immediate-early gene regulation is concerned. PDE10A was reported also to be mechanoresponsive in pulmonary arterial smooth muscle cells of a rat model for pulmonary hypertension. The inhibition of PDE10A with papaverine attenuated the effects on pulmonary hypertensive hemodynamic parameters and pulmonary vascular remodeling [31]. However, in hMSC, the upregulation of PDE10A itself by mechanical loading could not be blunted by papaverine treatment and stimulation with db-cAMP even increased the expression of PDE10A, indicating that mechanoresponsive upregulation of PDE10A itself is regulated by cAMP only when nonphysiological high concentrations are applied. Under physiological conditions, cAMP plays only a minor role, indicating that alternative second messengers and signaling pathways might be involved.

## 5. Conclusion

PDE10A is a relevant modulator of osteogenic differentiation in skeletal precursors. Specific PDE10A inhibition using papaverine diminishes osteogenic differentiation and in vitro mineralization. In addition, PDE10A gene expression is itself mechanoresponsive and its function modulates cellular mechanotransduction particularly with respect to immediate-early gene regulation. This paper extends the knowledge about the function of PDE10A in human bone marrow-derived stromal cells and osteogenic precursors. Future research will certainly unravel its role in the maintenance and adaptation of bone mass according to environmental needs in health and disease.

## Figures and Tables

**Table 1:** Primer names, sequences, product lengths, annealing temperatures, and GenBank Accession numbers are shown.

Gene name	Primer	Sequence 5′-3′	Product length	Annealing temp (°C)	GenBank accession number
Primers for human genes					
ALPL	ALPL_FOR	GTACGAGCTGAACAGGAACAACG	151	58	NM_000478.5
	ALPL_REV	CTTGGCTTTTCCTTCATGGTG			
RPS27A [37]	RPS27A_FOR	TCGTGGTGGTGCTAAGAAAA	141	60	NM_001135592
	RPS27A_REV	TCTCGACGAAGGCGACT			
RUNX2	Runx2_FOR	CTTCACAAATCCTCCCCAAG	147	58	NM_001024630.3
	Runx2_REV	ATGCGCCCTAAATCACTGAG			
SPP1	SPP1_FOR	TATGATGGCCGAGGTGATAG	133	60	NM_001040058.2
	SPP1_REV	CATTCAACTCCTCGCTTTCC			
Primers obtained from Qiagen					
PDE10A	Hs_PDE10a_1_SG	Qiagen sequence			NM_001130690; NM_006661
PTGS2	Hs_PTGS2_1_SG	Qiagen sequence		59	NM_000963
FOS	Hs_FOS_1_SG	Qiagen sequence		57	NM_005252.3
Primers for murine genes					
B2m	mB2m_FOR	GTCTTTCTGGTGCTTGTCTC	117	57	NM_009735.3
	mB2m_REV	AGTTCAGTATGTTCGGCTTC			
Pde10a	mPDE10A_FOR	TCTGAAAGTGTTAGTGCAGAGA	98	57	NM_001290707.1
	mPDE10A_REV	TGGTACCTGCTGACTTCCTT			
	mSPP1_REV	CGCTCTTCATGTGAGAGGTG			

## Data Availability

The raw data of the qPCR and viability assays used to support the findings of this study are available from the corresponding author upon request.

## Supplementary Materials

Supplementary materials Suppl. Figure 1: expression of PDE10A in primary hMSC after treatment with dexamethasone and papaverine.
