# Supplementary figures and images for: Phosphodiesterase 10A Is a Mediator of Osteogenic Differentiation and Mechanotransduction in Bone Marrow-Derived Mesenchymal Stromal Cells
Source: Stem Cells Int. 2020 Jun 6;2020:7865484. doi: 10.1155/2020/7865484 (PMC7294361; doi:10.1155/2020/7865484)

## Slide 1
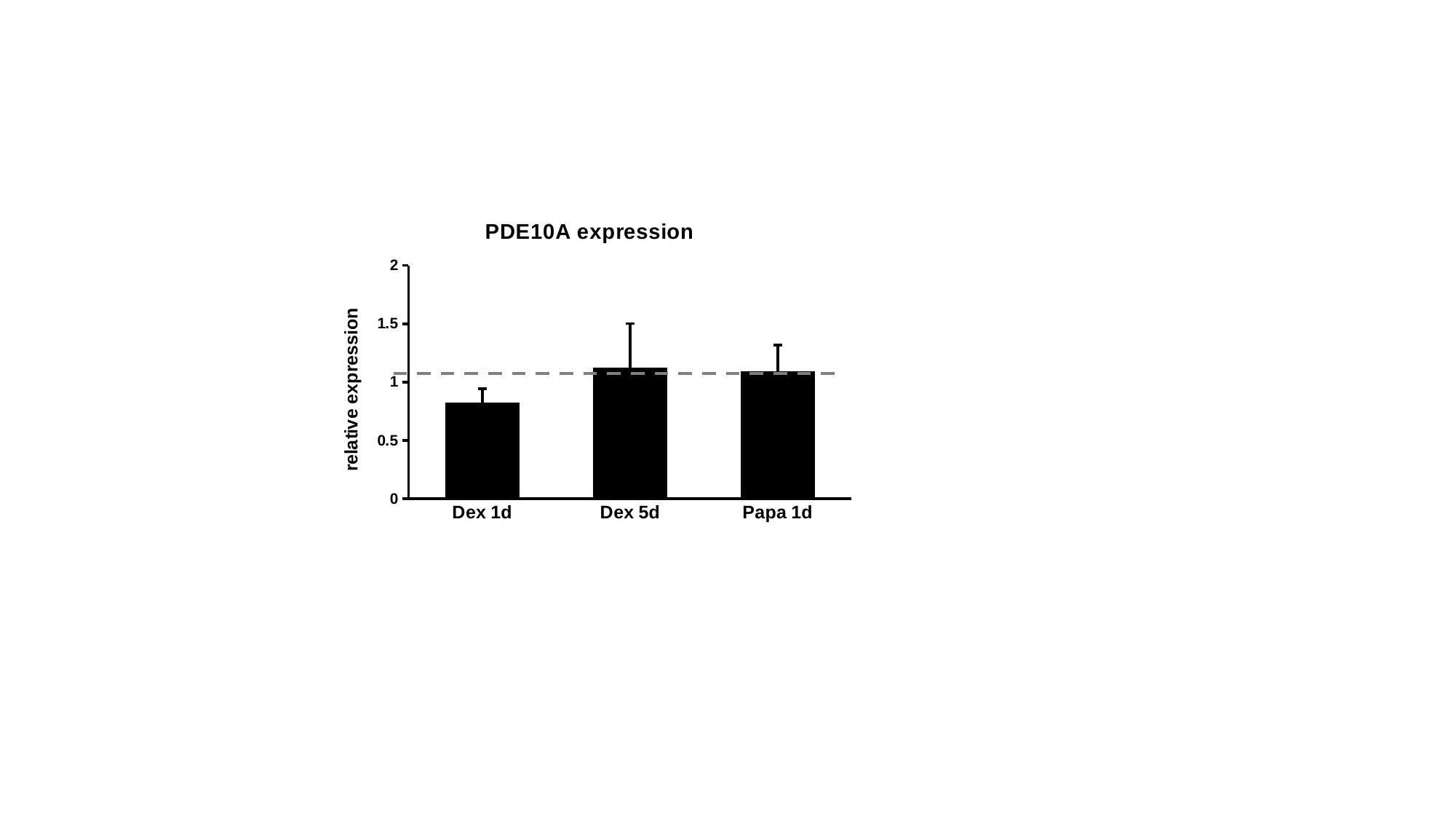

### Chart: PDE10A expression
| Category | |
|---|---|
| Dex 1d | 0.8181974842370486 |
| Dex 5d | 1.1195279466944041 |
| Papa 1d | 1.0881093356959979 |

Supplement: Supplementary materials — Suppl. Figure 1: expression of PDE10A in primary hMSC after treatment with dexamethasone and papaverine. [file 7865484.f1.pptx]
